# Peripheral blood transcriptomic differences predict depression status in individuals undergoing bariatric surgery

**DOI:** 10.1016/j.bbih.2025.101029

**Published:** 2025-06-09

**Authors:** Rebecca Milton, Anna P. McLaughlin, Nicole Mariani, Melisa Kose, Zuzanna Zajkowska, Giulia Lombardo, Naghmeh Nikkheslat, Esperanza Perucha, Valeria Mondelli

**Affiliations:** a Department of Psychological Medicine, King's College London, Institute of Psychiatry, Psychology and Neuroscience, London, United Kingdom; b Centre for Inflammation Biology and Cancer Immunology (CIBCI), Faculty of Life Sciences and Medicine, King's College London, London, United Kingdom; c National Institute for Health and Care Research (NIHR) Maudsley Biomedical Research Centre at South London and Maudsley NHS Foundation Trust and King's College London, London, United Kingdom

## Abstract

More than one third of candidates for bariatric surgery suffer with clinical depression. Significant reduction in depression following bariatric surgery has been shown, but this is not consistent for all patients. The biological mechanisms behind the association between obesity and depression and behind persistent/remitted depression post-surgery remain unclear. This study aimed to identify potential biological mechanisms involved in this association. As part of the longitudinal observational bariatric surgery and depression (BARIDEP) study, blood samples were collected from individuals prior to bariatric surgery. For this study we selected n = 29 subjects (from the original sample of n = 85 participants) based on their Hamilton Depression (HAM-D) scale scores at baseline and at 6 months post-surgery and grouped them as controls (n = 10), persistent depression (n = 7) or remission (n = 12). Participants were selected to be matched for age, sex and BMI. RNA was extracted and bulk RNAseq was performed. Data were analysed for differential expression and gene set enrichment across the 3 groups of interest. Analysis of the differential gene expression showed seven genes differentially expressed across the three groups, with genes mainly involved in immune activation or synaptic function. The greatest differences were found between the persistent and remitting depression groups, despite both experiencing clinical depression at the time of sample collection. Our data show distinct baseline gene expression and gene enrichment suggesting different immune and metabolic mechanisms possibly involved in persistent vs remitting depression post-bariatric surgery.

## 1 Introduction

Obesity and depression are growing medical and societal challenges, which often co-occur, forming a vicious circle where an individual is unable to recover from one condition while still experiencing the other. Understanding possible molecular mechanisms linking obesity and depression is important to reduce their comorbidity and improve clinical outcomes. One of the best naturalistic models to study biological mechanisms underlying comorbidity between depression and obesity is represented by individuals undergoing bariatric surgery. Bariatric candidates have increased rates of depression compared to the general population (Fitzgibbon et al., 1993; Alabi et al., 2018). Although the weight-loss following bariatric surgery is often associated with improvement of depressive symptoms and remission from depression, this association is not always reported, as some individuals still present with reduced weight loss and worse depression prognosis (Smith et al., 2020; Geerts et al., 2021). It remains so far unclear what biological pathways are involved in the persistence or remission of depression following bariatric surgery. A better understanding of these biological mechanisms is particularly important not only to identify at an early stage those individuals at risk of poor clinical outcome following surgery, but also to identify novel biological targets relevant for treatment strategies tackling comorbidity between depression and obesity.

Abnormalities in both energy regulating pathways and inflammation are some of the main mechanisms proposed to play a role in the comorbidity between obesity and depression (Castanon et al., 2014; Chan et al., 2019; Milano et al., 2020). More specifically, alterations in catabolic pathways due to mitochondrial dysfunction have been reported both in obesity (Bournat and Brown, 2010; Zheng et al., 2023) and in depression (Liu et al., 2023; Gu et al., 2021; Casaril et al., 2021; Bekhbat et al., 2021a). Similarly, increased levels of systemic inflammation are commonly found in both conditions (McLaughlin et al., 2021). Furthermore, our recent findings showed elevated peripheral inflammatory markers including C-Reactive Protein (CRP) and interleukin (IL)-6 in individuals with overweight/obesity and depression compared to individuals with these conditions alone (McLaughlin et al., 2021). In bariatric patients, the presence of increased systemic inflammation in those presenting with depression vs those without has also been reported (McLaughlin et al., 2023). Moreover, transcriptomic analyses in whole blood of 33 individuals undergoing bariatric surgery reported increased TP53 (Tumor Protein 53), GR (Glucocorticoid Receptor), and NFκB (Nuclear Factor kappa B) pathways associated with the presence of depression (Moisan et al., 2021). The follow-up of 24 of these patients confirmed these transcriptomic biomarkers as predictive of remission of depressive symptoms. However, this study did not interrogate biomarkers in individuals with persistent or worsening depression after bariatric surgery who do not see a decrease in depression symptoms alongside the decrease in weight and inflammation associated with bariatric surgery. Indeed, no other study has previously investigated biological differences between those individuals with remitted or persistent depression post-surgery. Further understanding of these groups of individuals may allow for elucidation of the mechanisms behind comorbid obesity and depression.

Genome-wide gene expression methods are particularly useful to understand altered transcriptomic pathways in disease in an unbiased way. Transcriptomics allow for the identification of novel pathways of interest and are able to look at larger gene sets than a targeted analysis would allow. In depression, previous studies have identified targets of therapeutic interest, particularly in treatment-resistant depression (Jansen et al., 2015; Bekhbat et al., 2021b; Amasi-Hartoonian et al., 2022).

**Fig. 1:**
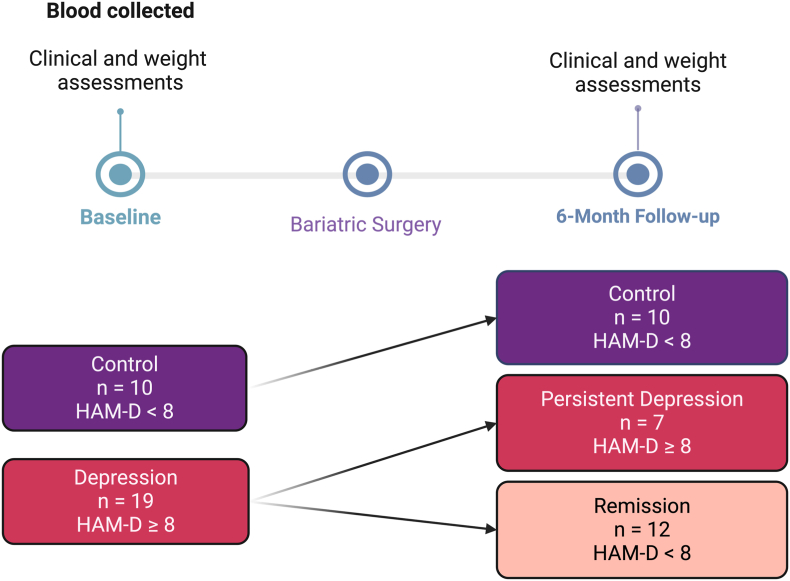
Study timeline and sample group classification. Schematic of the sample collection timeline and groupings for the BARIDEP participants selected for RNAseq analysis.

In this study we selected a genome wide expression approach to identify potential new pathways in comorbid obesity and depression at baseline, with two main aims: 1) to investigate differences in biological pathways between bariatric candidates with and without depression, and 2) to identify differences between subjects who experience persistent depression vs remission of depression. To address these aims we performed whole-blood bulk RNA-sequencing (RNAseq) from samples collected prior to bariatric surgery (baseline) in individuals who were assessed for presence/absence of depression at the time of sampling and 6-months following surgery (Fig. 1). Individual gene differences alongside gene set enrichment (GSEA) analyses were performed to identify novel mechanisms associated with persistence of depression.

## 2 Methods

### 2.1 Study information

This study is part of the bariatric surgery and depression (BARIDEP) project, an observational case-control longitudinal study conducted at King's College London, where participants were assessed before their bariatric surgery and followed up 6 months after surgery. The study was approved by the Research Ethics Committee (National Research Ethics Service East of England, Cambridge Central, UK; approval number: 18/LO/350). All procedures contributing to the study comply with the ethical standards of the relevant national and institutional committees on human experimentation and with the Helsinki Declaration of 1975, as revised in 2008. All participants provided written informed consent before entering the study.

### 2.2 Selection criteria for participants in this study

The original BARIDEP project recruited n = 85 subjects with obesity undergoing bariatric surgery, including 41 individuals with diagnosis of major depressive disorder (MDD) and 44 individuals without history of depression or severe mental health disorder at baseline assessment (before surgery). For the purpose of this study, we selected participants on the basis of their mental health outcome at 6-month post-surgery, using the severity of depressive symptoms measured with the Hamilton Depression Rating scale (HAM-D). Participants were divided into 3 groups: 1) control group (HAM-D < 8 at baseline and at follow-up), 2) persistent depression group (HAM-D > 8 at baseline and at follow-up), and 3) remission group (HAM-D > 8 at baseline and <8 at follow-up) (Fig. 1). Based on these criteria, individuals were selected from each group for RNAseq analyses (1 sample from the persistent depression group was later excluded from the analysis due to study ineligibility). The selection process of participants also included age, sex and BMI matching across the 3 groups (Table 1).

**Table 1:** Clinical and demographics characteristics of participants selected for RNAseq analysis. Abbreviations: SD = Standard deviation, BMI = body mass index, HAM-D = Hamilton Depression rating scale 17 for depressive symptoms.

Variable	Control (N = 10)	Depression (N = 7)	Remission (N = 12)	p-value^a^
Age (years)				0.6
Mean (SD)	45.4 (6.5)	45.3 (12.3)	48.4 (8.7)	
Gender (n (%))				0.9
Female	6 (60.0)	5 (71.4)	9 (75.0)	
Male	4 (40.0)	2 (28.6)	3 (25.0)	
Weight (Kg)				>0.9
Mean (SD)	137.4 (22.9)	133.4 (24.4)	136.6 (16.0)	
Body fat (%)				0.3
Mean (SD)	45.3 (2.5)	47.2 (3.8)	47.4 (4.4)	
Missing Data (n)			1	
Visceral Fat Rating				0.9
Mean (SD)	21.4 (7.8)	20.6 (6.6)	20.7 (5.3)	
Missing Data (n)			1	
BMI (Kg/mˆ2)				>0.9
Mean (SD)	47.9 (3.2)	48.6 (6.8)	48.7 (4.8)	
Antidepressant use (Baseline n(%))	0 (0.0)	5 (71.4)	8 (66.7)	<0.001
Antidepressant use (6M Follow-up, n (%))	0 (0.0)	3 (42.9)	7 (58.3)	0.009
HAM-D Score (Baseline)				<0.001
Mean (SD)	1.5 (1.8)	22.0 (5.9)	15.5 (3.9)	
HAM-D Score (6M Follow-up)				<0.001
Mean (SD)	0.9 (1.5)	20.9 (7.6)	3.4 (2.7)	

a Kruskal-Wallis rank sum test; Fisher's exact test.

There was not a statistically significant difference between the number of participants with mild, moderate or severe depression between the persistent depression and the remission group at baseline (p = 0.06, Fisher exact test).

As previously described (McLaughlin et al., 2023), participants were recruited from the bariatric surgery clinic at King's College Hospital. Eligibility criteria for all participants for the BARIDEP study were (1) obesity, based on a BMI ≥35, (2) scheduled to undergo either Roux-en-Y Gastric Bypass or Sleeve Gastrectomy bariatric surgery, (3) aged between 18 and 70 years old and (4) ability to read and write English fluently.

Eligibility criteria for participants with depression were a current diagnosis of major depressive disorder as defined by meeting criteria of the diagnostic and statistical manual of mental disorders, fifth edition (DSM-5) using the Mini International Neuropsychiatric Interview (MINI) (Sheehan, 1998). Eligibility criteria for control participants were absence of personal history of MDD or treatment with antidepressant medication for depressive symptoms and absence of lifetime history of severe mental illness.

Exclusion criteria for all participants for the BARIDEP study were (1) pregnancy or breastfeeding, (2) a past or current diagnosis of a psychotic disorder, (3) taking high doses of anti-inflammatory medication, (4) criteria met for alcohol abuse, drug abuse or dependence in the last 6 months, (5) participation in an investigational drug clinical trial within the last year, (6) a lifetime history of severe medical disorder or infection likely to compromise the interpretation of immunological data.

Depressive symptoms were measured at baseline prior to surgery and 6 months post-surgery using the structured interview guide of the Hamilton Depression Rating Scale (Hamilton, 1960) with Atypical Depression Supplement (SIGH-ADS) (Singh and Williams, 2006).

Body mass index (BMI) and weight were measured using the Tanita MC-780MA professional body composition analyser.

### 2.3 RNA sequencing analyses

Whole blood samples were collected into PAXgene RNA tubes (PreAnalytiX) at baseline visit, prior to bariatric surgery. Samples were rested at room temperature for 2 h (hrs), and stored at −20^o^C for 24hrs before being moved to long term storage at −80^o^C. RNA was isolated using the PAXgene RNA Isolation kit (Qiagen) according to manufacturer instructions and stored at −80^o^C prior to further processing.

Library preparation was conducted using the NEBNext Poly(A) mRNA Magnetic Isolation Module (NEB) followed by the NEBNext Ultra II Directional RNA Library Prep (NEB) according to manufacturer instructions. Sequencing was performed on the NovaSeq (Illumina) with >16 million reads per sample.

Raw FastQ files were quality checked using the FastQC program (Babraham Bioinformatics). Data were pseudoaligned to the Ensembl database (version GRCh38.p14) using the Kallisto pseudoaligner (Bray et al., 2016). Globin genes (HBA1, HBA2, HBB, HBBP1, HBD, HBE1, HBG1, HBG2, HBM, HBQ1, HBZ and HBZP1) were removed bioinformatically from the dataset to avoid skewness in the analysis (Sheerin et al., 2023). Genes with counts ≥ 10 in at least 7 samples were filtered for further analysis, resulting in 14933 genes for differential gene analysis.

Differential gene expression was performed using the DESeq2 R package (Love et al., 2014) with Benjamini Hochberg correction for multiple comparisons (Benjamini and Hochberg, 1995). Six-month follow-up and baseline depression status were used as the comparisons of interest in the design matrix to compare differentially expressed genes. The covariates also considered in the design matrix were library batch due to the presence of multiple randomised batches as well as gender of the participants due to uneven genders among groups. Significance of differentially expressed genes was determined by an adjusted P value (pAdj) < 0.1.

Gene set enrichment was performed in R using the clusterprofiler package (Yu et al., 2012) to the MsigDB Hallmark gene sets (Liberzon et al., 2015) using all genes sorted by Log2 fold-change (Log2FC). Significance was determined with an adjusted p value < 0.05.

Correlation analyses of gene counts alongside continuous demographic variables were performed using Spearman's rank correlation. Categorical differences of gene counts with gender or antidepressant use were determined using Wilcoxon rank-sum test or T test depending on data normality. Tests were performed in R and significance was defined as p < 0.05.

## 3 Results

### 3.1 Gene expression analyses between subjects with and without depression at baseline

**Fig. 2:**
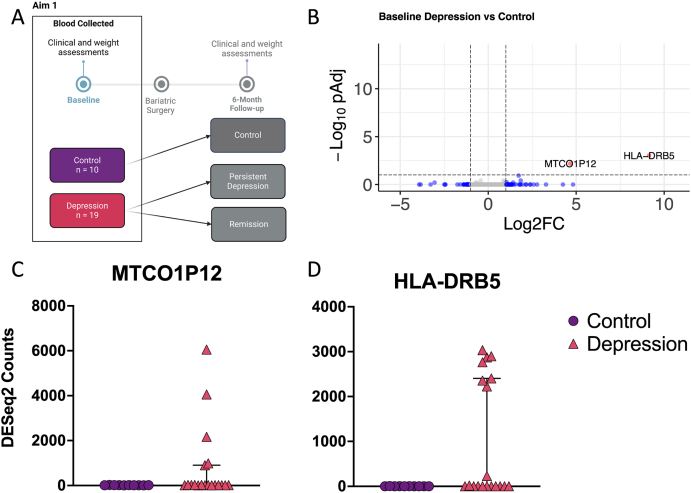
Baseline grouping differential gene expression. (A) Schematic of analysis strategy for aim 1. (B) Volcano plot of identified genes using DESeq2, highlighting in red genes with Log2FC > 1 and pAdj <0.1. Individual normalised gene counts for (C) HLA DRB5 and (D) MTCO1P12. Data show individual participant normalised DESeq2 counts for each gene. Bars show the median and interquartile range of each group.

To answer the first aim of our study, our analysis evaluated gene expression to determine differences between control and depression participants at baseline (Fig. 2A). Two genes, *MTC01P12* (Log2FC = 4.67, pAdj = 0.007) and *HLA-DRB5* (Log2FC = 9.14, pAdj = 0.001) were found to be significantly upregulated in the depression group when compared with controls (Fig. 2B). Observation of the individual normalised counts for each of these genes highlighted that the differential expression was driven by a subset of individuals, rather than the group as a whole (Fig. 2C and D). Due to the experimental model for analysis that was formulated to include library batch and gender as covariates, these differences could not have been driven by library batch of the samples or gender of the participants.

### 3.2 Gene expression analyses between subjects with persistent or remitted depression

**Fig. 3:**
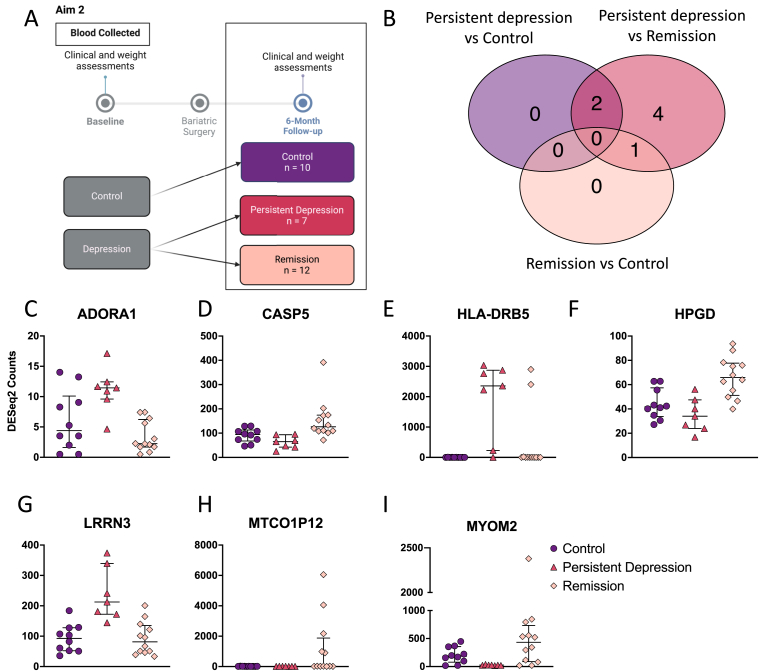
Follow-up group differential gene expression. (A) Schematic of analysis strategy for aim 2. (B) Numbers of differentially expressed genes determined by DESeq2 with pAdj <0.1. Individual normalised gene counts for (C) ADORA1 (D) CASP5 (E) HLA-DRB5, (F) HPGD, (G) LRRN3, (H) MTCO1P12 (I) MYOM2. Data show individual participant normalised DESeq2 counts. Bars show the median and interquartile range of each group.

At the 6-month follow-up visit, depression status was investigated. Here, the baseline depression group was divided between individuals that reached remission with HAM-D < 8 and individuals that were still experiencing depression defined by HAM-D ≥ 8. We interrogated our dataset to identify differences at baseline that could define these trajectories (Fig. 3A). This analysis strategy identified 7 genes differentially expressed among the 3 groups with pAdj <0.1 (Fig. 3B).

The analysis testing differences between the persistent depression and control groups highlighted 2 differentially expressed genes: *HLA-DRB5* (Log2FC = 11.23, pAdj = 5.38 × 10^−6^) and MYOM2 (Log2FC = −3.20, pAdj = 0.05) (Fig. 3E–I). *HLA-DRB5* was increased in the persistent depression group whereas *MYOM2* showed a decreased expression. When we tested for differences between the persistent and remitted depression groups, we found 7 differentially expressed genes: *ADORA1* (Log2FC = 2.36, pAdj = 0.06), *CASP5* (Log2FC = −1.27, pAdj = 0.03), *HLA-DRB5* (Log2FC = 9.39, pAdj = 9.97 × 10^−5^), *HPGD* (Log2FC = −0.94, pAdj = 0.08), *LRRN3* (Log2FC = 1.38, pAdj = 0.06), *MTCO1P12* (Log2FC = −5.73, pAdj = 5.49 × 10^−6^) and *MYOM2* (Log2FC = −4.14, pAdj = 5.49 × 10^−6^)(Fig. 3C–I). More specifically, expression of *HLA-DRB5* was again increased in the persistent depression group compared with remission alongside *ADORA1* and *LRRN3.* Expression of *CASP5*, *HPGD*, *MTCO1P12* and *MYOM2* were increased in the remission group compared with the persistent depression group. Interestingly, we only found 1 differentially expressed gene increased in the remission group when compared to control, *MTCO1P12* (Log2FC = 5.98, pAdj = 2.59 × 10^−8^) (Fig. 3D).

Of these genes, 2 genes (*HLA-DRB5* and *MYOM2*) were shared between the control *vs* persistent depression analysis and the persistent depression *vs* remission analysis. The gene *MTCO1P12* was shared between the control *vs* remission and the persistent depression *vs* remission analysis.

Data from the whole sample were tested for correlations with the key demographics age, BMI, body fat percentage and HAM-D score. Significant positive correlations were present between the HAM-D score and HLA-DRB5 (Supplementary Table 1, rho = 0.519, p = 0.004) as well as LRRN3 (rho = 0.479, p = 0.009). A significant positive correlation was also found between body fat percentage and HLA DRB5 (rho = 0.416, p = 0.028). There were no significant differences in gene counts between antidepressant use or gender in any of the genes of interest.

### 3.3 Gene Set Enrichment Analysis

**Fig. 4:**
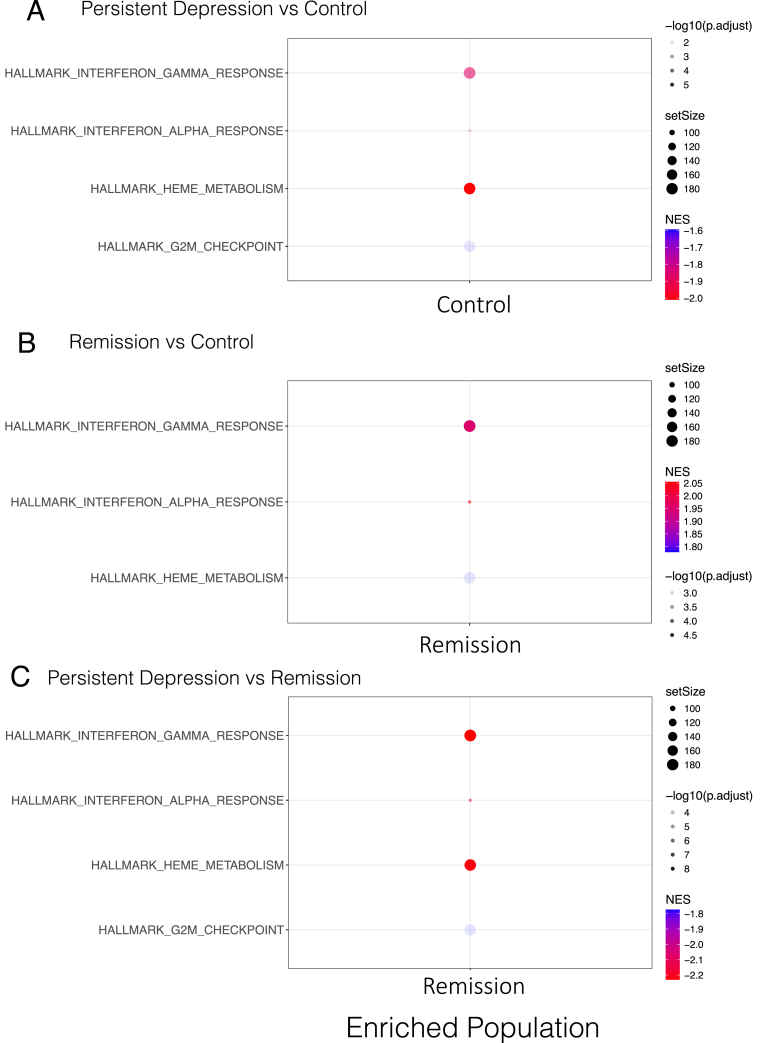
Follow up group gene set enrichment analysis. Gene Set Enrichment Analysis (GSEA) of (A) persistent depression vs control, (B) remission vs control and (C) persistent depression vs remission comparisons. The enriched population is the group which had a significant enrichment of each gene set within each group comparison. In each of the plots, circle size represents the gene set size of the enriched population, circle transparency represents the -log10 adjusted p value for the enrichment, circle colour represents the normalised enrichments score (NES) scaled from the most (red) to the least (blue) enrichment for each comparison.

As individual gene changes cannot often capture larger systemic alterations within heterogeneous sample sets, we performed gene set enrichment analysis of the Hallmark gene sets using the 3 follow-up group comparisons (Fig. 4). Comparison between control and persistent depression groups showed an enrichment of the heme metabolism, interferon gamma response, interferon alpha response and G2M checkpoint gene sets in the control group (Fig. 4A). In contrast, comparison between control and remission groups (Fig. 4, B) or between persistent depression and remission groups (Fig. 4, C) showed an enrichment of the heme metabolism, interferon gamma response and interferon alpha response gene sets in the remission group. The remission group was also enriched for the G2M checkpoint gene set when compared with the control group.

## 4 Discussion

In this study, we aimed at dissecting, at the molecular level, differences between persistent and remittent depression in obese individuals. Our data confirms the presence of blood transcriptomic differences between obese individuals with and without depression prior to bariatric surgery and validate previous reports. Moreover, our findings are the first to identify significant differences in transcriptomic signatures involved in immune activation and synaptic function between individuals with persistent depression over time when compared to those who achieve remission 6-months after bariatric surgery. Lastly, our study shows an increased enrichment for genes belonging to the Heme metabolism pathway, particularly in individuals who experience remission of depression following bariatric surgery.

The increased expression of *HLA-DRB5* identified in patients with depression vs controls appears mainly driven by patients with persistent depression. HLA-DRB5 is part of the major histocompatibility complex class 2, involved in antigen presentation to CD4^+^ T cells, a type of adaptive immune cell at the centre of the inflammatory response. Interestingly, its increased expression has been previously associated with other neuroinflammatory disorders such as multiple sclerosis (Enz et al., 2020; Lang et al., 2002), Parkinson's disease (Nalls et al., 2019) and Alzheimer's disease (Lambert et al., 2013) as well as with remission to depression with antidepressants in late life depression (Eyre et al., 2016). Our findings may therefore suggest that antigen presentation, CD4^+^ T cell activation and/or other immune pathway related to the expression of HLA-DRB5 would therefore potentially be less sensitive to the “antidepressant effect” of bariatric surgery.

Among the other 6 genes which we found differentially expressed among groups, *ADORA1, MYOM2* and *LRRN3* have also been previously described in the literature as being associated with depression. These genes appeared particularly associated with the persistent depression state in our study. *MYOM2* encodes the myomensin-2 protein. While it is particularly associated with cardiovascular disease, it was also found to be downregulated in individuals with MDD compared with healthy controls prior to any anti-depressant treatment (Singh et al., 2024). Our data also showed a downregulation in those with persistent depression and no difference in those with remitting depression. This may indicate possible mechanistic similarities between those with persistent depression following bariatric surgery and those with depression alone, in contrast with individuals with remitting depression who do not share the downregulation of this gene. *LRRN3* is involved in synaptic assembly and has been previously shown to be downregulated in the cerebral cortex of juvenile rats treated with antidepressant drugs (Tsapakis et al., 2014) and increased in individuals with childhood trauma (Cattaneo et al., 2018). The *ADORA1* gene encodes the adenosine A1 receptor. This has previously been part of a gene set of RELA target genes associated with depression prior to bariatric surgery (Moisan et al., 2021). While the previous study did not see changes in *ADORA1* directly, in our cohort *ADORA1* was increased in the persistent depression group only and not in the remitting group. This is in agreement with the previous findings where the gene set was higher in individuals with MDD. While the function and expression of *LRRN3* and *ADORA1* have been associated with brain function, we were able to detect differences in the blood transcriptome, with transcripts showing higher expression in the persistent depression group when compared with control and remission. Of note, gene expression in blood and brain samples has shown to have some level of correlation (Cai et al., 2010; Sullivan et al., 2006), possibly indicating that these changes may either be representative of alterations happening in the brain or evidence of signals inducing transcriptomic changes in the brain that may be acting in the periphery in a similar way.

Our gene set enrichment analyses data show an enrichment in the Heme metabolism genes, particularly in the remission group. Heme is a bioavailable form of iron synthesised in the mitochondria and degraded by heme oxygenase (HO-1), leading to the release of free iron into circulation (Dutt et al., 2022). Heme metabolism products, alongside with HO-1, have been shown to have immunomodulatory effects (Ryter and Choi, 2016), lead to neuronal apoptosis through astrocytes during oxidative stress (Yang et al., 2017), and cause systemic inflammation in a mouse model of inflammation induced depression-like behaviour (Ma et al., 2023). Moreover, decreased HO-1 levels in the serum are associated with depressive symptoms in individuals with hypertension (Robaczewska et al., 2016). Previous studies have also reported associations between changes in circulating iron and depression (Shariatpanaahi et al., 2007; Yi et al., 2011). Increased cytokines, which have been shown to be present in the participants with depression in this study (McLaughlin et al., 2023), can also lead to decreased circulating iron through increased secretion of hepcidin (Nemeth et al., 2004). On the other hand, the presence of differential enrichment in these pathways in our study may also be an indicator of mitochondrial metabolic differences between the control and persistent depression/remission groups. The heme metabolism pathway is tightly regulated and occurs mostly within the mitochondria (Yien and Perfetto, 2022). This is further supported by the mitochondrial pseudogene *MTCO1P12* having increased expression in the remission group. *MTCO1P12* has also been seen to be increased in PBMCs of individuals with bipolar disorder (Qi et al., 2023) and decreased in post-mortem spinal cord of individuals with Amyotrophic Lateral Sclerosis (Ziff et al., 2023). Mitochondrial dysfunction has also been implicated in depression leading to increased inflammation and production of reactive oxygen species (Khan et al., 2023).

The identification of gene set enriched between obese individuals with and without depression prior to bariatric surgery is consistent with findings from a previous transcriptomic study (Moisan et al., 2021). Although the individual genes we describe herein do not overlap with those from this previous study, both studies point towards activation of similar immune pathways, such as IFN signalling (Moisan et al., 2021), as shown by our GSEA. These pathways have also been identified in other transcriptomic studies researching inflammatory depression (Cho et al., 2019). Interestingly, our study shows that these differences may be driven by a particular subset of patients more likely to present with remission of depression following bariatric surgery.

The majority of gene set enrichment differences were found not between the control and persistent depression groups, as we would have anticipated, but between the persistent depression and remission groups. The remission group showed the highest enrichment for these pathways, suggesting that individuals in this group may be experiencing more immunometabolic dysregulation than the persistent depression individuals. While the participants of each group did not show differences in their clinical presentation or severity of depression at the time of sampling, it is striking that they were different transcriptomically, both at the individual gene and at the gene set level, even before undergoing bariatric surgery. Our transcriptomic data further suggests that while increased inflammation is present in all of the patients with depression at this timepoint, the mechanisms producing the inflammation may differ between the remission and persistent depression patients that results is poorer short term mental health outcomes following bariatric surgery. Transcriptomically, those with persistent depression display an immune activation linked to HLA dysregulation and synaptic signalling, while those going into remission presenting with an immune activation more associated with abnormalities in energy regulating pathways/mitochondrial function.

Our study was limited by the small number of participants in each group, that could have hindered the identification of gene differences and pathways due to lack of statistical power. However, these pilot data have allowed for the identification of differences amongst the groups and highlights the importance for looking at depression outcome following surgery when exploring depression in individuals undergoing bariatric surgery. Furthermore, our sample was strengthened by the *a priori* selection of the participants, ensuring similar age, sex and BMI among the groups, and supporting that the differences identified are independent to gene expression differences caused by obesity. An additional limitation comes from our inability to correct for cell-type within our data; however, to the best of our knowledge, there is an absence of reliable datasets that can be used as bulk RNASeq references for distinct cell types within whole blood. We also did not correct for other covariates such as antidepressant use and body fat percentage due to the lack of antidepressant use within the control group and missing data within the body fat percentage data. However, there was no significant difference between the 2 groups with depression in terms of their antidepressant use and there was no statistically significant difference in body fat percentage amongst the 3 groups. Finally, although this is a longitudinal study, our findings cannot make firm conclusions about causative effects of these genes or pathways and mechanistic preclinical studies would need to be performed to clarify causation vs association effects.

In conclusion, we provide further evidence of inflammation and metabolism related pathways being important in depression outcomes in individuals with obesity. Our study highlights the presence of transcriptomic differences suggesting different biological/immune pathways involved in persistence and remission of depression in individuals undergoing bariatric surgery.

## CRediT authorship contribution statement

**Rebecca Milton:** Writing – review & editing, Writing – original draft, Visualization, Investigation, Formal analysis, Data curation, Conceptualization. **Anna P. McLaughlin:** Writing – review & editing, Investigation, Conceptualization. **Nicole Mariani:** Writing – review & editing, Investigation. **Melisa Kose:** Writing – review & editing, Investigation. **Zuzanna Zajkowska:** Writing – review & editing, Investigation. **Giulia Lombardo:** Writing – review & editing, Investigation. **Naghmeh Nikkheslat:** Writing – review & editing, Investigation. **Esperanza Perucha:** Writing – review & editing, Supervision, Funding acquisition, Conceptualization. **Valeria Mondelli:** Writing – review & editing, Supervision, Funding acquisition, Conceptualization.

## Declaration of competing interest

The authors declare the following financial interests/personal relationships which may be considered as potential competing interests: Co-author is a member of the BBI-Health editorial board - V.M. If there are other authors, they declare that they have no known competing financial interests or personal relationships that could have appeared to influence the work reported in this paper.

## Data Availability

Data will be made available on request.
