# Supplementary material for: Peripheral blood transcriptomic differences predict depression status in individuals undergoing bariatric surgery
Source: Brain Behav Immun Health. 2025 Jun 9;47:101029. doi: 10.1016/j.bbih.2025.101029 (PMC12205765; doi:10.1016/j.bbih.2025.101029)
Supplement: Multimedia component 1 [file mmc1.docx]

Supplementary table 1: Correlation analyses of genes of interest with continuous demographic variables

| Gene | Demographic | S | P value | Rho |
| --- | --- | --- | --- | --- |
| HLA-DRB5 | Baseline Hamd17 | 1953.7 | 0.003933 | 0.5187968 |
|  | Baseline BMI | 3289.8 | 0.3243 | 0.1896973 |
|  | Baseline Body Fat Percentage | 2134.3 | 0.02772 | 0.4158962 |
|  | Age | 4619.2 | 0.4762 | -0.1377221 |
| MTCO1P12 | Baseline Hamd17 | 3799.9 | 0.7413 | 0.06406986 |
|  | Baseline BMI | 5163.8 | 0.1537 | -0.2718758 |
|  | Baseline Body Fat Percentage | 2874 | 0.2741 | 0.2134647 |
|  | Age | 3433.3 | 0.424 | 0.1543584 |
| ADORA1 | Baseline Hamd17 | 3181.8 | 0.2597 | 0.2163115 |
|  | Baseline BMI | 3787.7 | 0.7295 | 0.06707768 |
|  | Baseline Body Fat Percentage | 4390.4 | 0.3038 | -0.2015334 |
|  | Age | 4388.5 | 0.6765 | -0.08091774 |
| CASP5 | Baseline Hamd17 | 4487.8 | 0.5864 | -0.1053813 |
|  | Baseline BMI | 3277.4 | 0.3164 | 0.1927533 |
|  | Baseline Body Fat Percentage | 4118 | 0.518 | -0.1269841 |
|  | Age | 3473.3 | 0.4546 | 0.1444952 |
| HPGD | Baseline Hamd17 | 3468.4 | 0.4508 | 0.1457033 |
|  | Baseline BMI | 3690.7 | 0.6389 | 0.09095393 |
|  | Baseline Body Fat Percentage | 3214 | 0.5401 | 0.120416 |
|  | Age | 3554.4 | 0.5198 | 0.1245223 |
| LRRN3 | Baseline Hamd17 | 2116.6 | 0.008619 | 0.4786687 |
|  | Baseline BMI | 4293.2 | 0.7673 | -0.05743162 |
|  | Baseline Body Fat Percentage | 2786 | 0.2226 | 0.2375479 |
|  | Age | 4528.5 | 0.5511 | -0.1153989 |
| MYOM2 | Baseline Hamd17 | 5062.3 | 0.1967 | -0.2468792 |
|  | Baseline BMI | 3959.9 | 0.899 | 0.02464876 |
|  | Baseline Body Fat Percentage | 2816 | 0.2394 | 0.2293377 |
|  | Age | 3031.9 | 0.185 | 0.2532365 |

Supplementary table 2: Comparative analysis of categorical demographics with genes of interest

| Gene | Demographic | p value | stat | Test |
| --- | --- | --- | --- | --- |
| HLA-DRB5 | AD | 0.117 | 69 | Wilcoxon Rank-Sum Test |
|  | Gender | 0.171 | 118.5 | Wilcoxon Rank-Sum Test |
| MTCO1P12 | AD | 0.682 | 94 | Wilcoxon Rank-Sum Test |
|  | Gender | 0.116 | 124 | Wilcoxon Rank-Sum Test |
| ADORA1 | AD | 0.809 | 98 | Wilcoxon Rank-Sum Test |
|  | Gender | 0.268 | 66 | Wilcoxon Rank-Sum Test |
| CASP5 | AD | 0.531 | 89 | Wilcoxon Rank-Sum Test |
|  | Gender | 0.365 | 70 | Wilcoxon Rank-Sum Test |
| HPGD | AD | 0.876 | -0.1583611 | T Test |
|  | Gender | 0.563 | 0.5916759 | T Test |
| LRRN3 | AD | 0.374 | 83 | Wilcoxon Rank-Sum Test |
|  | Gender | 0.234 | 116 | Wilcoxon Rank-Sum Test |
| MYOM2 | AD | 0.983 | 103 | Wilcoxon Rank-Sum Test |
|  | Gender | 0.295 | 113 | Wilcoxon Rank-Sum Test |
